# Comment on: “Probiotics in addition to antibiotics for the treatment of acute tonsillitis: a randomized, placebo-controlled study”

**DOI:** 10.1007/s10096-015-2377-y

**Published:** 2015-04-21

**Authors:** F. Di Pierro

**Affiliations:** Scientific Department, Velleja Research, Milan, Italy

Carried out by: P. Gilbey, L. Livshits, A. Sharabi-Nov, Y. Avraham, D. Miron.

Published in: European Journal of Clinical Microbiology & Infectious Diseases

doi:10.1007/s10096-015-2315-z

Gilbey et al. report on the results of a double-blind placebo-controlled trial carried out to investigate the benefits of the *Streptococcus salivarius* K12 probiotic usage while undergoing antibiotic treatment for acute pharyngotonsillitis. The results of their study showed no significant difference between either treatment group (probiotic or placebo) when used in conjunction with intravenous penicillin, for any of the parameters assessed during the acute phase of the disease. This result is not surprising considering that the probiotic used is already well known to be sensitive to penicillin [1–3], and, therefore, is likely to be killed before it is able to exert its protective effect. Since the efficacy of penicillin for the treatment of acute group A *Streptococcus* (GAS) pharyngitis is well described, it would be difficult to envision a scenario where the *S. salivarius* probiotic could enhance the clearance rate of GAS and reduce the time required for recovery.

In their justification for carrying out this investigation, the authors cite examples of probiotics that have reduced the duration of acute respiratory tract infections in support of their trial design [4]. However, these were carried out without the complication of co-treatment with antibiotics; therefore, it is not clear whether the benefits would still have been observed if antibiotics were a component of the treatment regimes [4]. Another example cited by the authors demonstrated a benefit from the concomitant administration of antibiotics, and an antibiotic-resistant probiotic for reducing the recurrence rates of urinary tract infections in children [5]. Again, this is a different trial design when compared to that carried out by Gilbey et al., as it is not investigating the effect on acute symptoms of the disease.

A more informative trial assessing the benefits of K12 during acute infection would be to investigate the downstream effect of K12 when used in conjunction with penicillin, and continued post-antibiotic therapy, for the prevention of secondary infections or recurrences of GAS. Previous studies have clearly demonstrated that daily consumption of *S. salivarius* K12 can reduce GAS pharyngotonsillitis recurrence rates by up to 90 % [6–8], and this would surely be worth investigating as a potential positive outcome from a co-treatment regime, before recommending that K12 should not be combined with antibiotics.

Therefore, with the considerations above, the recommendation of the study’s authors not to combine *S. salivarius* K12 with antibiotic treatment for acute pharyngitis cases seems premature. While an immediate benefit related to pain scores and immune marker levels may not have been observed, combined treatment, particularly as an adjunct therapy that extends beyond the period of antibiotic dosing, is likely to still yield significant benefits for the patient when the downstream effects are considered, compared to antibiotic treatment alone.
